# EFFECTS OF HOME-BASED COGNITIVE AND PHYSICAL EXERCISE TRAINING ON COGNITION IN OLDER ADULTS: THE COVEPIC TRIAL

**DOI:** 10.1093/geroni/igae098.2186

**Published:** 2024-12-31

**Authors:** Louis Bherer, Caroll-Ann Blanchette, Florent Besnier, Mathieu Gayda, Christine Gagnon, Thomas Vincent, Tudor Vrinceanu, Emma Dupuy

**Affiliations:** University of Montreal, Montreal, Quebec, Canada; Montreal Heart Institute, Montréal, Quebec, Canada; Montreal Heart Institute, Montréal, Quebec, Canada; Université de Montréal, Montreal, Quebec, Canada; Montreal Heart Institute, Montréal, Quebec, Canada; Montreal Heart Institute, Montréal, Quebec, Canada; Montreal Heart Institute, Montréal, Quebec, Canada; Montreal Heart Institute, Montréal, Quebec, Canada

## Abstract

**Background:**

Studies suggest that cognitive training and physical exercise can independently help improve cognition in older adults. This randomized clinical trial (COVEPIC study) aimed to compare the effects of 6 months of home-based physical exercise alone and combined with cognitive training on the cognition of older adults.

**Methods:**

127 adults (50 years and older) were randomly assigned to one of the two following intervention arms (1:1): 1/ home-based physical exercise alone or 2/ combined home-based physical exercise and cognitive training. Participants completed videoconference neuropsychological assessments targeting episodic and working memory, processing speed and executive functions, prior to the intervention (T0), mid-intervention (T1) (3 months), and post-intervention (T2) (6 months). Clinicaltrials.gov (NCT04635462).

**Results:**

Participation in the combined intervention showed a larger improvement than the exercise alone group on the MoCA from T0 to T1, and a change in executive functions which was not observed in the physical exercise only group. Effects of exercise dose were also observed, as participants who went through a higher dose of exercise showed larger improvement in episodic and working memory.

**Discussion:**

Results suggest that a home-based intervention combining cognitive and physical training can help improve cognition in older adults.

